# Association of intraoperative cell salvage combined with standard allogeneic transfusion with hematological parameters and clinical outcomes in pediatric tracheoplasty: a non-randomized cohort study

**DOI:** 10.3389/fped.2026.1755494

**Published:** 2026-08-03

**Authors:** Yuejiao Han, Lili Su, Xiaoya Ye, Yuanqing Hua, Changbin Zhao, Wenxuan Liu, Ying Qiu

**Affiliations:** 1 Department of Anesthesiology, Children’s Hospital of Shandong University (Jinan Children’s Hospital), Jinan, China; 2 Department of Urology, Children’s Hospital of Shandong University (Jinan Children’s Hospital), Jinan, China; 3 Department of Cardiac Surgery, Children’s Hospital of Shandong University (Jinan Children’s Hospital), Jinan, China

**Keywords:** allogeneic transfusion, children, coagulation function, hematological indicators, intraoperative cell salvage

## Abstract

**Objective:**

To investigate associations of intraoperative autologous blood salvage (IOCS) combined with allogeneic transfusion vs. allogeneic transfusion alone in pediatric slide tracheoplasty (STP) for congenital tracheal stenosis (CTS), regarding hematological parameters, coagulation function, clinical outcomes, and safety.

**Methods:**

From October 2023 to February 2025, 69 children meeting inclusion criteria were enrolled in this non-randomized study. Based on parental consent for IOCS, 35 were allocated to the observation group (IOCS + allogeneic transfusion) and 34 to the control group (allogeneic transfusion alone). Pre- and postoperative hematological and coagulation parameters, allogeneic transfusion volume, adverse reactions, postoperative fever, hospital stay, and 6-month mortality were compared.

**Results:**

Preoperative indicators showed no significant intergroup differences (*P* > 0.05). Postoperatively, the observation group had higher red blood cell count, hematocrit, and hemoglobin (all *P* < 0.05); shorter prothrombin time and higher fibrinogen (both *P* < 0.05); and no significant differences in APTT or TT (both *P* > 0.05). Intraoperative allogeneic red blood cell and fresh frozen plasma volumes were significantly lower in the observation group (both *P* < 0.05). No hemolytic or allergic transfusion reactions occurred. Postoperative fever was higher in the control group (*P* < 0.05), and hospital stay was shorter in the observation group (*P* < 0.05). No deaths occurred within 6 months.

**Conclusion:**

IOCS combined with allogeneic transfusion was associated with better postoperative hematological and coagulation profiles, reduced allogeneic blood requirements, and shorter hospital stay. No transfusion-related adverse reactions or deaths were observed. These findings warrant confirmation in prospective randomized studies.

## 1 Introduction

Congenital tracheal stenosis (CTS) is characterized by relative narrowing resulting from hypoplasia or malformation of the tracheal cartilage. In CTS, the normal membranous posterior tracheal wall (including smooth muscle) is replaced by cartilage, leading to the conversion of the typical C-shaped cartilaginous rings into complete O-shaped rings. CTS is a severe congenital airway malformation that can be life-threatening in children. It typically manifests in early infancy (1), is rare, and is associated with a poor prognosis; more than two-thirds of affected children have concomitant congenital heart disease (CHD) (2). CTS with CHD represents one of the most challenging conditions in pediatric cardiac surgery, as it adversely affects perioperative prognosis and increases mortality in children with CHD. Once diagnosed, surgical intervention should be performed as early as possible. Tracheoplasty is a primary treatment option and can achieve long-term survival rates exceeding 88% (1, 3). However, given the prolonged operative time, extensive surgical trauma, potential vascular injury, substantial blood loss, and perioperative coagulation disturbances, children may experience significant perioperative hemorrhage and have limited tolerance to hypovolemia, which can readily result in impaired peripheral perfusion (4). Therefore, blood transfusion is critical for maintaining hemodynamic stability during surgery. Currently, allogeneic transfusion is the mainstream perioperative blood support strategy for pediatric tracheoplasty, and all children enrolled in this study received standard allogeneic packed red blood cells and auxiliary blood products when clinically necessary. As a complementary blood conservation technique, intraoperative autologous cell salvage (IOCS) can be combined with routine allogeneic transfusion to reduce perioperative allogeneic blood exposure. Compared with allogeneic banked blood, reinfused salvaged red blood cells have higher viability, greater oxygen-carrying capacity, minimal immunomodulatory effects, and a lower incidence of allergic reaction (5). Their biochemical properties more closely resemble those of endogenous blood, thereby reducing the risk of transfusion-related complications. IOCS is now widely used in procedures with anticipated substantial blood loss (6). This study compared IOCS combined with allogeneic transfusion vs. allogeneic transfusion alone in pediatric STP to provide evidence for clinical blood management.

## 2 Objects and methods

### 2.1 General information

This prospective non-randomized cohort study was approved by the Ethics Committee of Jinan Children's Hospital (No. SDFE-IRB/P-2023047). Sixty-nine children undergoing STP were enrolled.

Inclusion Criteria were as follows: (1) a confirmed diagnosis of CTS with CHD; (2) anticipated intraoperative blood loss > 500 mL or > 10% of the patient’s estimated total blood volume; (3) written informed consent obtained from a parent/legal guardian, including consent for intraoperative blood salvage (IOCS);(4) American Society of Anesthesiologists (ASA) physical status classification II–IV.

Exclusion criteria were as follows: (1) severe cardiac insufficiency/heart failure, diagnosed hematologic diseases, or known coagulation disorders; (2) severe renal insufficiency or requirement for hemodialysis; (3) a history of radiotherapy, chemotherapy, or immunosuppressant therapy; (4) sepsis or septicemia; (5) a history of transfusion-related complications.

All patients received standard allogeneic transfusion during surgery. Patients were divided into observation group (IOCS combined with standard allogeneic transfusion, *n* = 35) and control group (standard allogeneic transfusion alone, *n* = 34) according to parental consent for IOCS. Baseline data were comparable between groups (*P* > 0.05). (Table 1).

**Table 1:** Comparison of general data between the two groups of children ($\bar{X}$ ±S).

Indicator	Control Group (*n* = 34)	Observation Group (*n* = 35)	P
Age (years)	2.67 ± 2.08	3.67 ± 2.31	0.067
Weight (kg)	13.14 ± 8.02	16.48 ± 10.00	0.131
Gender			
Male	24 (70.59)	19 (54.29)	0.162
Female	10 (29.41)	16 (45.71)	
ASA Classification			
Class II	3 (8.82)	1 (2.86)	0.504
Class III	24 (70.59)	24 (68.57)	
Class IV	7 (20.59)	10 (28.57)	

### 2.2 Methods

#### 2.2.1 Anesthesia procedure

Children in both groups underwent standard preoperative fasting (6 h for solids and 4 h for clear liquids). Two or more intravenous access lines were established before transfer to the operating room. On arrival, vital signs were continuously monitored, including oxygen saturation (SpO₂), blood pressure (BP), heart rate (HR), electrocardiogram (ECG), and temperature.

No premedication was administered before anesthesia induction. Tracheal intubation was performed during induction while preserving spontaneous respiration. Induction was achieved with 3%∼5% sevoflurane delivered via a face mask combined with intravenous propofol (1∼2 mg·kg⁻^1^). An appropriately sized endotracheal tube was inserted orally under direct visualization using a video laryngoscope. Correct tube placement was confirmed with a fiberoptic bronchoscope, after which cisatracurium (0.15∼0.2 mg·kg⁻^1^) and sufentanil (0.1∼0.5 μg·kg⁻^1^) were administered intravenously.

Once spontaneous respiration ceased, pressure-controlled ventilation (PCV) was initiated. Inspiratory pressure was titrated to achieve a tidal volume (VT) of 6∼8 mL·kg⁻^1^. Ventilator settings were as follows: fraction of inspired oxygen concentration (FiO₂) 0.5∼0.8, oxygen flow 2 L·min⁻^1^, inspiration-to-expiration ratio (I:E) 1:1.5-2, and respiratory rate (RR) 25∼50 breaths·min⁻^1^.

Anesthesia Maintenance: Anesthesia was maintained with continuous infusions of propofol (8∼12 mg·kg⁻^1^·h⁻^1^) and remifentanil (0.2∼0.3 μg·kg⁻^1^·min⁻^1^). Infusions were discontinued during cardiopulmonary bypass (CPB) and restarted after aortic unclamping, and continued until the end of surgery.

After intubation, a radial artery catheter was performed for continuous blood pressure monitoring and arterial blood gas analysis. An internal jugular venous catheter was inserted for central venous access and central venous pressure (CVP) monitoring. Nasopharyngeal and rectal temperatures were monitored continuously throughout the procedure. Additional intravenous boluses of cisatracurium and sufentanil were administered as needed.

Before initiating CPB and after aortic unclamping, ventilator settings were adjusted to maintain end-tidal carbon dioxide (PetCO₂) at 35∼45 mmHg. Intravenous fluid type and volume were titrated according to vital signs, hemodynamic parameters (HR, MAP, CVP), and urine output (>0.5 mL·kg⁻^1^·h⁻^1^). Vasoactive agents were administered as necessary to maintain hemodynamic stability, defined as heart rate (HR) and mean arterial pressure (MAP) within 20% of baseline values, and a CVP of 5–12 cm H₂O.

At the end of surgery, all children were transferred intubated to the Cardiac Intensive Care Unit (CICU) under continued anesthesia.

#### 2.2.2 Surgery and cardiopulmonary bypass

Pediatric patients who met the inclusion and exclusion criteria underwent surgery performed by relatively consistent surgical and CPB teams. The CPB circuit was primed with crystalloid, colloid, and other solutions. Systemic anticoagulation was achieved with heparin; and CPB was initiated via arterial and venous cannulation after the activated clotting time (ACT) reached the target level.

#### 2.2.3 Transfusion methods

The transfusion protocol was developed in accordance with the Expert Consensus on Clinical Pathway Management for Autologous Blood Transfusion (2019) issued by the Working Group of the Clinical Transfusion Committee of the Chinese Society of Blood Transfusion. The protocol applies to surgical patients with an estimated recoverable blood volume exceeding 1,000 mL (a threshold that may be appropriately lowered in pediatric patients given their smaller total blood volume) or to cardiac surgery patients who meet the indications for autologous blood salvage. All patients in both groups received standard allogeneic blood transfusion support based on clinical intraoperative needs. Specifically, the control group received routine allogeneic transfusion alone throughout the operation without intraoperative autologous blood salvage and reinfusion. The observation group received intraoperative autologous blood salvage and reinfusion on the basis of standard routine allogeneic transfusion: salvaged red blood cells were prioritized for intraoperative transfusion, and supplementary allogeneic red blood cells, plasma, and cryoprecipitate were administered timely when the autologous blood volume was insufficient or clinical transfusion indications were met.

#### 2.2.4 IOCS method

Intraoperative autologous blood transfusion was performed using a Cell Saver 5 + device. Preoperative setup included preparation of the anticoagulant solution (25,000 U heparin in 500 mL normal saline), a reservoir, a suction device (negative pressure 120–150 mmHg), connecting tubing, a centrifuge bowl set, and a wash solution. The tubing and reservoir were primed with 150 mL of anticoagulant, after which the anticoagulant infusion rate was adjusted to approximately 20 drops/min. Blood salvage was initiated at the start of surgery. Blood loss from the operative field following skin incision was aspirated into the reservoir. Blood was not salvaged during tracheal repair or reconstruction procedures (excluding cases of acute massive hemorrhage). Blood from the surgical field was continuously suctioned into the reservoir. After discontinuation of cardiopulmonary bypass, residual blood in the circuit tubing was also aspirated into the reservoir. When the collected blood reached 400 mL or clinical transfusion was needed, the blood was processed via filling, centrifugation and washing, and the concentrated red blood cells were transferred into a collection bag. When transfusion was indicated, the processed blood was rapidly reinfused through a disposable leukocyte filter (Haemonetics Corporation - SQ40SK). Pressurized infusion was strictly prohibited (7).

#### 2.2.5 Observation indicators

Clinical data were extracted retrospectively from the HIS system and the anesthesia management system. Collected data included: (1) patient characteristics: sex, age, and ASA classification; (2) hematological parameters and postoperative allogeneic transfusion requirements: red blood cell (RBC) count, hematocrit (Hct), hemoglobin (Hb) level;and, for each allogeneic blood product [allogeneic red blood cells, fresh frozen plasma (FFP), platelets, and cryoprecipitate], both the number of patients who received the product and the transfusion volume in each group; (3) coagulation parameters: thromboelastography (TEG)–based coagulation assessment, including prothrombin time (PT), activated partial thromboplastin time (APTT), thrombin time (TT), and fibrinogen (FIB); and (4) outcome measures: incidence of transfusion reactions, postoperative fever, length of hospital stay (LOS), and 6-month postoperative mortality.

### 2.3 Statistical methods

Statistical analyses were performed using SPSS software (version 29.0). Continuous variables with normal distributions were presented as the mean ± standard deviation (x¯ ± s) and compared between groups using the independent-samples t-test. Measurement data with non-normal distribution were analyzed using the rank-sum test (Mann–Whitney U test). Categorical variables were presented as numbers (%) [n (%)] and were compared using the Pearson *χ*^2^ test or Fisher's exact test. To minimize potential selection bias and confounding from non-random assignment, analysis of covariance (ANCOVA) was used to compare postoperative hematological indices between groups, adjusted for preoperative baseline levels. Postoperative indices were dependent variables; group (IOCS + allogeneic transfusion vs. allogeneic transfusion alone) was the fixed factor, and preoperative values were covariates. All tests were two-tailed, and *P* values < 0.05 were considered statistically significant.

## 3 Results

### 3.1 Comparison of hematological and coagulation indices between the Two groups

Postoperative hematological and coagulation indices were compared using ANCOVA adjusted for preoperative baseline values. Preoperatively, no statistically significant differences were observed between the two groups in red blood cell count, hematocrit, and hemoglobin (all *P* > 0.05). After adjustment, the observation group demonstrated superior blood indices compared to the control group. Regarding coagulation, the observation group had a significantly shorter prothrombin time (PT) and higher fibrinogen (FIB) levels than the control group (both *P* < 0.05). There were no statistically significant differences in thrombin time (TT) between groups. Although the between-group difference in activated partial thromboplastin time (APTT) did not reach statistical significance, APTT tended to be shorter in the observation group (Table 2).

**Table 2:** Comparison of hematological and coagulation indicators between the two groups of children ($\bar{X}$ ±S).

Indicator	Group	Preoperative	Postoperative	Estimated value	95%CI	P
RBC Count (×10^12^/L)	Control Group (*n* = 34)	4.47 ± 0.46	4.39 ± 0.52	4.38 ± 0.12	4.137∼4.624	0.001*
	Observation Group (*n* = 35)	4.34 ± 0.58	4.97 ± 0.86	4.98 ± 0.12	4.742∼5.223	
Hematocrit (L/L)	Control Group (*n* = 34)	37.08 ± 3.91	37.41 ± 3.38	37.36 ± 0.76	35.85∼38.88	0.041*
	Observation Group (*n* = 35)	36.54 ± 4.27	39.56 ± 5.25	39.59 ± 0.75	38.09∼41.09	
Hemoglobin (HGB) (g/L)	Control Group (*n* = 34)	121.08 ± 12.56	125.29 ± 11.23	125.27 ± 2.32	120.64∼129.91	0.046*
	Observation Group (*n* = 35)	120.45 ± 15.34	131.89 ± 15.28	131.90 ± 2.28	127.33∼136.46	
PT(s)	Control Group (*n* = 34)	11.96 ± 0.94	14.04 ± 2.31	14.05 ± 0.31	13.42∼14.68	0.011*
	Observation Group (*n* = 35)	12.02 ± 0.99	12.89 ± 1.29	12.88 ± 0.31	12.26∼13.51	
APTT(s)	Control Group (*n* = 34)	29.54 ± 4.38	28.45 ± 7.92	28.25 ± 1.01	26.23∼30.26	0.066
	Observation Group (*n* = 35)	28.62 ± 4.03	25.40 ± 3.52	25.59 ± 0.99	23.61∼27.58	
TT(s)	Control Group (*n* = 34)	17.78 ± 1.38	15.64 ± 1.65	15.67 ± 0.24	15.19∼16.15	0.638
	Observation Group (*n* = 35)	18.14 ± 1.63	15.85 ± 1.11	15.83 ± 0.24	15.36∼16.30	
FIB(g/L)	Control Group (*n* = 34)	2.36 ± 0.44	3.50 ± 1.13	3.51 ± 0.21	3.09∼3.92	0.014*
	Observation Group (*n* = 35)	2.44 ± 0.72	4.24 ± 1.27	4.24 ± 0.21	3.83∼4.65	

* Significant difference at *p* < 0.05.

### 3.2 Comparison of postoperative transfusion volumes between the Two groups

#### 3.2.1 Allogeneic red blood cells

All 34 patients (100%) in the control group received allogeneic RBC transfusion, vs. 35 of 35 patients (100%) in the observation group. The mean volume transfused was higher in the control group than in the observation group (1.31 ± 1.04 U vs. 0.91 ± 0.45 U; *P* < 0.05).

#### 3.2.2 Fresh frozen plasma (FFP)

All 34 patients (100%) in the control group received FFP, vs. 35 of 35 patients (100%) in the observation group. The mean volume of FFP transfused was higher in the control group (257.35 ± 196.99 mL vs. 181.43 ± 90.82 mL; *P* < 0.05).

Platelets: No patient in either group received a platelet transfusion [0/34 [0%] in the control group; 0/35 [0%] in the observation group].

#### 3.2.3 Cryoprecipitate

Cryoprecipitate was administered to 6 of 34 patients (17.65%) in the control group and 4 of 35 patients (11.43%) in the observation group; the difference was not statistically significant (*χ*^2^ = 0.132, *P* = 0.716). Among recipients, the mean volume transfused was 1.32 ± 0.41 U in the control group and 1.25 ± 0.37 U in the observation group (U = 566.00, *P* = 0.530). Full details are presented in Table 3.

**Table 3:** Comparison of postoperative blood transfusion volume between Two groups of children ($\bar{X}$ ±S).

Variable	Control Group Recipients(n)	Control Group	Observation Group Recipients(n)	Observation Group	t/U	P
Allogeneic Red Blood Cells(U)	34/34	1.31 ± 1.04	35/35	0.91 ± 0.45	2.074	0.042*
Fresh Frozen Plasma(mL)	34/34	257.35 ± 196.99	35/35	181.43 ± 90.82	2.066	0.043*
Platelet(U)	0/34	0	0/35	0	-	-
Cryoprecipitate(U)	6/34	1.32 ± 0.41	4/35	1.25 ± 0.37	566.000	0.530

* Significant difference at *p* < 0.05.

### 3.3 Comparison of adverse reactions and hospital stay duration between the Two groups

No hemolytic or allergic transfusion reactions were observed in either group. Postoperative fever occurred in 13 children (37.14%) in the control group and in 5 children (14.71%) in the observation group, with a statistically significant difference between groups (*P* < 0.05). The mean length of hospital stay was 37.31 ± 25.91 days in the control group and was significantly shorter in the observation group (26.41 ± 11.27 days; *P* < 0.05). No deaths occurred in either group during the study period (Table 4).

**Table 4:** Comparison of incidence of reactions and length of hospital stay between Two groups [n(%)，($\bar{X}$ ±S)].

Group	Hemolytic Reaction	Allergic Reaction	Postoperative Fever	Length of Hospital Stay (days)	Number of Deaths
Control Group (*n* = 34)	0 (0.00)	0 (0.00)	13 (37.14)	37.31 ± 25.91	0 (0.00)
Observation Group (*n* = 35)	0 (0.00)	0 (0.00)	5 (14.71)	26.41 ± 11.27	0 (0.00)
X^2^*/t*	-	-	4.503	2.255	-
P	-	-	0.034*	0.027*	-

* Significant difference at *p* < 0.05.

## 4 Discussion

This was a non-randomized cohort study, and group allocation was based on parental/guardian consent rather than randomization, which may introduce selection bias and confounding. Baseline characteristics were well balanced, and statistical adjustments were applied to reduce confounding; however, residual confounding could not be completely excluded.

Congenital tracheal stenosis is a rare but life-threatening condition, and tracheoplasty remains a pivotal intervention for improving survival in affected children. Previous studies have shown that most patients are infants and young children whose hematopoietic function is not fully mature, further increasing operative complexity and posing substantial clinical challenges (8, 9). Given the escalating risks and costs associated with blood transfusion, perioperative blood management is increasingly important to minimize blood loss and optimize red blood cell utilizatio (10). Different from the simple autologous vs. allogeneic transfusion comparison in previous studies, all subjects in this study received routine allogeneic transfusion as the basic blood support strategy. The core difference between the two groups lies in the application of intraoperative autologous blood salvage on the basis of standard allogeneic transfusion. Compared with the single routine allogeneic transfusion strategy, the combined strategy of autologous blood salvage plus allogeneic transfusion offers better blood component compatibility, avoids excessive allogeneic blood exposure and related complications such as immunosuppression and transfusion-related acute lung injury, which is particularly suitable for children with immature immune systems (11). In addition, autologous blood salvage was associated with lower total allogeneic blood product requirements, which may relieve the pressure of clinical blood supply, and reduce the incidence of transfusion-related medical disputes. Although preoperative equipment preparation may increase upfront costs, this combined blood management strategy was associated with lower rates of allogeneic transfusion-related complications and postoperative treatment costs, suggesting potential long-term economic benefits (12).

### 4.1 Impact of intraoperative autologous blood salvage combined With standard allogeneic transfusion on postoperative hematological parameters and allogeneic transfusion requirements in pediatric patients

In the observation group, the mean volume of reinfused autologous blood was 190.43 ± 172.61 mL. Between-group comparisons showed that children receiving intraoperative autologous blood salvage combined with allogeneic transfusion had more favorable postoperative hematological parameters, including higher red blood cell counts and hemoglobin levels, than those receiving allogeneic transfusion alone. Furthermore, postoperative requirements for allogeneic red blood cells and fresh frozen plasma were lower in the observation group. These findings are consistent with a report on blood salvage in cardiac surgery (13). Previous studies have also demonstrated that adjuvant autologous blood salvage during cardiac surgery was associated with improve postoperative hemoglobin levels and reduce reliance on allogeneic blood transfusion (14). Intraoperative autologous blood reinfusion may supplement intraoperative red blood cell loss and improve systemic oxygen-carrying capacity, thereby potentially reducing the demand for supplementary allogeneic blood products (15, 16). Moreover, because salvaged blood primarily consists of red blood cells, it does not introduce large amounts of additional components such as coagulation factors or platelets, thereby minimizing interference with anticoagulation management during cardiopulmonary bypass. International studies further suggest that Cell Saver technology, as an effective adjuvant blood conservation measure, may provide clinical benefits for complex, prolonged pediatric surgical procedures with anticipated substantial blood loss (17).

### 4.2 Impact of intraoperative autologous blood salvage combined With standard allogeneic transfusion on postoperative coagulation parameters in children

In the comparison of coagulation parameters, postoperative PT was shorter, and FIB levels were higher in the observation group (IOCS combined with standard allogeneic transfusion) than in the control group (standard allogeneic transfusion alone). This phenomenon is related to the characteristics of IOCS blood processing. The centrifugation and washing steps of cell salvage can cause mild blood cell damage and remove excess heparin, free hemoglobin, and tissue debris in the circulating blood, which may help optimize the postoperative coagulation state on the basis of routine allogeneic transfusion.

Although the washing process eliminates a certain proportion of plasma coagulation factors, the reduction of allogeneic blood infusion volume in the observation group offsets this deficiency. FIB, the most abundant coagulation factor in plasma, directly participates in clot formation and acts as an acute-phase reactant elevated by surgical stress. The results indicated that the combined transfusion strategy was associated with better maintenance of postoperative coagulation function compared with allogeneic transfusion alone. It is still necessary to dynamically monitor coagulation indicators during clinical application to achieve individualized blood transfusion management (18).

### 4.3 Association between intraoperative autologous blood salvage combined With standard allogeneic transfusion and clinical outcomes in children

No hemolytic or allergic transfusion reactions occurred in either group during the perioperative period. The significantly lower incidence of postoperative fever in the observation group indicates that intraoperative cell salvage was not associated with increased postoperative inflammatory responses. The higher fever rate in the control group is probably related to surgical trauma and the immune-inflammatory reaction induced by allogeneic blood products. Within the observed sample, the combination of IOCS and allogeneic transfusion appeared to be associated with a lower incidence of perioperative adverse events; however, the absence of hemolytic/allergic reactions or deaths in either group limits formal safety comparisons, and conclusions about the safety profile should be limited to the observed data. Consistent with this finding, previous studies have reported that this combined blood management model was associated with lower allogeneic blood utilization and perioperative adverse event rates (19). In terms of clinical recovery efficiency, the combined transfusion group had a significantly shorter length of hospital stay. The supplementary transfusion of high-quality autologous salvaged red blood cells improves early postoperative oxygen delivery and tissue perfusion, accelerates postoperative physical functional recovery, and thus shortens the hospitalization cycle on the basis of routine allogeneic blood support (20). No deaths occurred in either group during the 6-month postoperative follow-up, which is reassuring, although the small sample size and the absence of adverse events in both groups preclude definitive conclusions about comparative long-term safety. Notably, the combined transfusion strategy was associated with favorable clinical outcomes and fewer transfusion-related adverse events in the observed sample (21).

### 4.4 Limitations and prospects

First, this was a non-randomized cohort study with group allocation based on parental/guardian consent, which may lead to selection bias and confounding. Although baseline balance and statistical adjustment were used to mitigate these issues, the non-randomized design prevents definitive causal conclusions, and this represents a major limitation of the study. Second, as a single-center study with a small sample size and a short follow-up duration, it was unable to assess the long-term effects of combined autologous and allogeneic transfusion strategy on hematological recovery or postoperative growth and development. Future multicenter studies with larger cohorts and longer follow-up are warranted. In addition, incorporating evaluations of red blood cell function, immune-related markers, and cost-effectiveness would help further clarify the long-term efficacy and safety of this combined blood transfusion strategy.

Beyond tracheoplasty, the combined strategy of intraoperative cell salvage plus routine allogeneic transfusion may also be extended to other major pediatric procedures associated with substantial blood loss—such as surgery for congenital heart disease and scoliosis correction—particularly in children with anticipated blood loss exceeding 10% of total blood volume. With ongoing advances in cell-salvage technology and optimization of individualized combined transfusion protocols, this dual blood management strategy has the potential to become a cornerstone of pediatric perioperative blood management, providing stronger support for perioperative safety and improved clinical outcomes.

## Data Availability

The raw data supporting the conclusions of this article will be made available by the authors, without undue reservation.

## References

[1] Sidell DR Meister KD de Alarcon A Boudewyns A Brigger M Chun R. International pediatric otolaryngology group (IPOG) consensus recommendations: evaluation and management of congenital tracheal stenosis. Int J Pediatr Otorhinolaryngol. (2022) 161:111251. 10.1016/j.ijporl.2022.111251 35988373

[2] McCarthy JF Hurley JP Neligan MC Wood AE. Surgical relief of tracheobronchial obstruction in infants and children. Eur J Cardiothorac Surg. (1997) 11(6):1017–22. 10.1016/S1010-7940(97)01166-4 9237581

[3] Wen W Du X Zhu L Wang S Xu Z Lu Z. Surgical management of long-segment congenital tracheal stenosis with tracheobronchial malacia. Eur J Cardiothorac Surg. (2022) 61(5):1001–10. 10.1093/ejcts/ezab551 34940823

[4] Al-Ammouri I Karasneh S Samara D Al-theiabat M Khriesat WM. Angioplasty of native coarctation in a very low birth weight, donor of twin-twin transfusion infant. Pediatr Cardiol. (2022) 43(2):467–9. 10.1007/s00246-021-02752-5 34655297

[5] Frank SM Sikorski RA Konig G Tsilimigras DI Hartmann J Popovsky MA. Clinical utility of autologous salvaged blood: a review. J Gastrointest Surg. (2020) 24(2):464–72. 10.1007/s11605-019-04374-y 31468332

[6] Waters JH. Intraoperative blood recovery. Asaio J. (2013) 59(1):11–7. 10.1097/MAT.0b013e31827b5187 23232181

[7] Tanh N Thach PN Phong LS Ngoc LTD Nguyen PT Van Anh Dung H. Tracheal reconstruction surgery for congenital tracheal stenosis. Pediatr Surg Int. (2023) 39(1):123. 10.1007/s00383-023-05418-w 36787049

[8] Hofferberth SC Watters K Rahbar R Fynn-Thompson F. Evolution of surgical approaches in the management of congenital tracheal stenosis: single-center experience. World J Pediatr Congenit Heart Surg. (2016) 7(1):16–24. 10.1177/2150135115606627 26714989

[9] Spahn DR Munoz M Klein AA Levy JH Zacharowski K. Patient blood management. Effectiveness and Future Potential. Anesthesiology. (2020) 133(1):212–22. 10.1097/ALN.0000000000003198 32108683

[10] Feuer G Katiyar P Reyes JL Taber C Coury JR Hassan FM. Intraoperative cell salvage utilization for blood conservation during pediatric and adult spinal surgery: a state-of-the-art systematic review. Spine Deform. (2025) 13(5):1305–26. 10.1007/s43390-025-01098-9 40465098

[11] Gao Y Chen X Mei Y Yang T Huang X Zhang H. Evaluation of staged autologous blood transfusion during extracorporeal membrane oxygenation decannulation: a retrospective study. Heart Lung. (2024) 68:202–7. 10.1016/j.hrtlng.2024.07.009 39043085

[12] Mello M Pereira-Rufino L Santos AA Junior NAH Panfilio CE de Souza AS. Evaluation of intraoperative and postoperative blood cell salvage use in cardiac surgery with cardiopulmonary bypass. Braz J Cardiovasc Surg. (2025) 40(3):e20240244. 10.21470/1678-9741-2024-0244 40445084 PMC12124749

[13] Lindsey P Echeverria A Poi MJ Matos J Bechara CF Cheung M. Thromboembolic risk of endovascular intervention for lower extremity deep venous thrombosis. Ann Vasc Surg. (2018) 49:247–54. 10.1016/j.avsg.2017.10.004 29197610

[14] Quispe-Fernandez LA Carrillo-Gonzalez ML Gutierrez-Ospina A Gómez-Leandro II. Blood products with autologous transfusion versus allogeneic transfusion in cardiac surgery patients. Rev Med Inst Mex Seguro Soc. (2020) 58(4):417–27. 10.24875/RMIMSS.M20000066 34543547

[15] Wang G Bainbridge D Martin J Cheng Davy. The efficacy of an intraoperative cell saver during cardiac surgery: a meta-analysis of randomized trials. Anesth Analg. (2009) 109(2):320–30. 10.1213/ane.0b013e3181aa084c 19608798

[16] Reyes G Prieto M Alvarez P Orts M Bustamante J Santos G. Cell saving systems do not reduce the need of transfusion in low-risk patients undergoing cardiac surgery. Interact Cardiovasc Thorac Surg. (2011) 12(2):189–93. 10.1510/icvts.2010.251538 21118833

[17] Adam EH Funke M Zacharowski K Meybohm P Keller H Weber CF. Impact of intraoperative cell salvage on blood coagulation factor concentrations in patients undergoing cardiac surgery. Anesth Analg. (2020) 130(5):1389–95. 10.1213/ANE.0000000000004693 32058448

[18] Liu Y Li X Che X Zhao G Xu M. Intraoperative cell salvage for obstetrics: a prospective randomized controlled clinical trial. BMC Pregnancy Childbirth. (2020) 20(1):452. 10.1186/s12884-020-03138-w 32767971 PMC7412832

[19] Miao Z Liu D Chu Z Zheng T Li B Liu P. Intraoperative cell salvage reduces postoperative allogeneic blood transfusion and shortens off-bed time in simultaneous bilateral total hip arthroplasty: a single-center retrospective study. BMC Musculoskelet Disord. (2024) 25(1):685. 10.1186/s12891-024-07807-1 39217321 PMC11365131

[20] Li W Li J Zhang X Li W Li Q Zhang X. Effect of intraoperative blood salvage autotransfusion on the prognosis of patients after carotid body tumor resection. Beijing Da Xue Xue Bao Yi Xue Ban. (2025) 57(2):272–6. 10.19723/j.issn.1671-167X.2025.02.008 40219556 PMC11992442

[21] Wu WW Zhang WY Zhang WH Yang Lei Deng XQ Ou MC. Survival analysis of intraoperative blood salvage for patients with malignancy disease: a PRISMA-compliant systematic review and meta-analysis. Medicine (Baltimore). (2019) 98(27):e16040. 10.1097/MD.0000000000016040 31277097 PMC6635293

